# The Long-Term Effects of Postoperative Radiotherapy in Acromegaly: Results From a Single-Center Acromegaly Registry in Iran

**DOI:** 10.1155/ije/9945510

**Published:** 2025-11-24

**Authors:** Farzad Najafipour, Javad Jafarpour, Amir Bahrami, Majid Mobasseri, Mitra Niafar, Mostafa Najafipour, Akbar Aliasgarzadeh, Naser Aghamohammadzadeh, Jalil Houshyar, Vahideh Sadra, Naimeh Mesri Alamdari

**Affiliations:** Endocrine Research Center, Tabriz University of Medical Sciences, Tabriz, Iran

**Keywords:** acromegaly, conventional radiotherapy, growth hormone, IGF-1, radiotherapy

## Abstract

**Background:**

Acromegaly is a rare but severe condition characterized by the excessive secretion of growth hormone (GH), leading to various metabolic alterations. Treatment options include surgery, pharmacotherapy, and radiotherapy (RT). RT can control the disease biochemically, either alone or in conjunction with medical therapy. This study aimed to evaluate the effectiveness of conventional RT in patients with postoperative acromegaly over a 15-year follow-up period, using specific cutoff criteria.

**Methods:**

A retrospective analysis was performed on 55 acromegaly patients who were monitored for an average of 15 (13.3–16.7) years and treated with conventional RT (median dose of 52 Gy) following pituitary surgery. Hormonal assessments included baseline and glucose-suppressed GH and IGF-1 levels, and side effects associated with RT were evaluated.

**Results:**

The baseline GH level decreased from 20.7 (16–25.4) μg/L to 11.2 (8.3–14.1) μg/L (*p* < 0.001) at 2 years, further dropping to 5.8 (4.99–6.61) μg/L (*p* < 0.001) at 5 years, and to 2.2 (1.66–2.74) μg/L (*p* < 0.001) at 10 years after RT. Rates of patients achieving suppressed GH levels < 1 μg/L rose from 9% at 2 years to 25% at 5 years, 42% at 10 years, and 76% at 15 years. IGF-1 levels normalized in 5% of participants at 2 years, 14% at 5 years, 38% at 10 years, and 60% at 15 years. After 10 years, 78% of patients experienced hypogonadism, 80% experienced hypothyroidism, and 82% experienced hypocortisolism. Neurological complications were reported in 4% of patients 10 years post-RT, while 4% developed visual impairments and optic neuropathy within 5 years.

**Conclusion:**

The results suggest that conventional RT is an effective long-term management strategy for patients who do not respond adequately to medical therapy or surgery. However, the high incidence of late-onset hypopituitarism must be taken into account.

## 1. Introduction

Acromegaly is a rare and advanced endocrine disorder that poses significant health risks, primarily due to pituitary adenomas, with less common causes including pituitary hyperplasia or ectopic secretion of growth hormone (GH) or GH-releasing hormone (GHRH) [1–4]. Prolonged excessive hormone exposure results in complications such as facial and acral overgrowth, prognathism, hyperhidrosis, goiter, sleep apnea, osteoarthritis, reproductive issues, and metabolic and cardiovascular diseases, adversely affecting quality of life and survival rates [5–7]. The disorder typically progresses unnoticed, leading to delayed diagnosis [8–10].

Management focuses on controlling GH and IGF-1 levels, alleviating symptoms, and reducing morbidity and mortality [11–14]. The primary treatment is neurosurgery for tumor removal, deemed the best first-line therapy for acromegaly [12, 15]. However, complete resection rates for macroadenomas are often low, particularly for large, invasive tumors. Consequently, an estimated 40%–50% of patients require second-line therapies, including pharmacological or radiotherapeutic options [16]. These second-line treatments include dopamine agonists (DAs) like cabergoline (CAB), GH receptor antagonists like pegvisomant (PEG), and somatostatin analogs (SSAs) such as lanreotide and octreotide [2]. The high cost of pharmacological treatments poses challenges, especially in low-income countries, where availability and tolerability can also be issues [17, 18].

Recently, stereotactic radiosurgery (SRS) has attracted attention as a second- or third-line treatment option for acromegaly [19]. SRS delivers higher doses of radiation in a three-dimensional manner, targeting tumors while minimizing risks such as hypopituitarism, radiation-induced optic neuropathy (RION), and secondary tumors [20]. However, careful patient selection is required to avoid excessive exposure to the optic apparatus [21].

Research on SRS indicates lower remission rates compared to traditional radiotherapy (RT), but it may result in a faster reduction of GH levels with fewer side effects. Conventional RT remains vital when other treatments fail, often employed for patients who are ineligible for surgery or pharmacotherapy [19, 22, 23]. Each treatment has specific benefits and limitations, highlighting the need for a combined approach to effectively manage acromegaly [24].

RT has been shown to improve hormonal parameters in about 60% of patients and effectively regulate tumor growth [25]. However, there are ongoing debates regarding its long-term efficacy in acromegaly [24]. Follow-up of patients who failed surgery and underwent RT indicated that 46% and 57% achieved GH levels below 1 μg/L at 5 and 10 years, respectively, while normal IGF-1 levels were reached in 36% and 43% at those time points [26]. Another study found that 22% of patients achieved GH levels under 2.5 ng/mL by 2 years, with 60% at 10 years and 77% at 20 years [27]. Nonetheless, the effectiveness of pituitary irradiation remains contested, as evidenced by Cozzi et al., who reported that only 16% of patients had normal IGF-1 levels after 10 years, with GH levels < 2.5 μg/L in 12% after 9 years [28]. While RT positively affects GH levels, its impact on IGF-1 is negligible, with only 6% achieving normal IGF-1 levels after a mean follow-up of 6.8 years [29, 30].

Complications such as necrosis and optic neuropathy are significant concerns [28, 31]. Long-term recovery may take 10–15 years, requiring ongoing pharmacotherapy to manage GH and IGF-1 levels [32]. In developing countries, due to the limited availability and cost-effectiveness of neurosurgery and pharmacotherapy, conventional RT is often the preferred treatment option. Despite its established efficacy in hormonal reduction, long-term outcomes of RT remain underresearched.

Currently, there are no studies examining the effectiveness of postoperative RT in acromegaly patients in Iran. Therefore, the present study aims to analyze single-center registry data regarding the effects of conventional radiation therapy on GH and IGF-1 secretion, as well as potential side effects and complications, using recent precise criteria cutoffs [31, 33].

## 2. Materials and Methods

### 2.1. Study Design and Population

A retrospective analysis was conducted from the IRAN acromegaly Registry system in 55 patients with acromegaly referred to the endocrinology clinic of Imam Reza Hospital, Tabriz, Iran, from 2006 to 2021. Between 2006 and 2021, 120 patients with acromegaly underwent transsphenoidal or transfrontal microsurgery in the Imam Reza Hospital, Tabriz Medical Center. Acromegaly was determined based on the characteristic clinical features of acromegaly and confirmed by insufficient suppression of GH value during GTT, and most had evidence of a tumor remnant on magnetic resonance imaging (MRI). Fifty-five of the 120 operated patients received adjuvant RT for residual disease and were selected for further analysis. In the remaining 65 patients, RT was the first choice, either alone (27 patients) or followed by surgical resection (38 patients).

The information in the database involves the patient demographics, clinical and biochemical presentation, and histology of operated tumors. The study participants include patients who had received RT as the treatment approach after surgical resection of a pituitary adenoma by transsphenoidal or transfrontal approach. Active acromegaly was distinguished through the generic clinical signs of the disorder and simultaneous shortage of GH suppression to < 1 ng/mL after a 75-g oral glucose load and the high age-adjusted IGF-1 values.

Clinical and biochemical examinations of decreased hormone concentrations were conducted to diagnose hypopituitarism as a side effect of RT. Gonadotrophin deficiency was determined as low or normal values of LH and FSH levels in postmenopausal women, in reproductive-aged women with amenorrhea, or low plasma testosterone concentrations in men (< 2·5 ng/mL) [33]. Central hypothyroidism was determined based on the low FT4 levels with low or normal TSH levels. Adrenal inadequacy was defined as low morning cortisol and/or subnormal cortisol in response to synthetic ACTH. Neurological events were determined through computed tomographic scans and/or MRI.

Informed consent was obtained from all subjects and/or their legal guardian(s).

This investigation was authorized by the Ethics Committee of Tabriz University of Medical Sciences with grant number 71046 and ethics code IR.TBZMED.REC.1401.932. All methods were performed in accordance with the Declaration of Helsinki guidelines and regulations.

### 2.2. Eligibility Criteria

The important inclusion criteria include age over 18 years and a known case of acromegaly (through tests and clinical symptoms). Key exclusion criteria included pregnancy, thyroid nodular diseases, presence of malignancy and other underlying diseases, withdrawal during the study period, not using the RT, not taking the prescribed medications, and lack of data in follow-up.

### 2.3. RT Protocol

The RT was applied through the lineal accelerator at an average total dose of 52 Gray units (49–60) delivered as 2–2.5 Gy daily administration 5 days a week. All patients were treated at the Imam Reza Hospital, Tabriz Radiation Therapy Center. Different treatment methods were conducted at the radiation therapy center. Multiple beam energies were applied: 1.75 megavolt (MV) g-rays from a ^60^Co source in the first years of the research, including 2006–2015, and 5, 15-, or 25-MV X-rays provided by the linear accelerator in the latter.

The aim volume was determined through lateral and anterior simulation films instructed by radiological and surgical discoveries and with contrast-enhanced computed tomography scans to conformal treatment planning procedures. Patients were treated with mixture of three coplanar fields (two lateral and one anterior), and more rarely a fourth posterior field was applied to spare temporal lobes.

### 2.4. Hormonal Assays

Biochemical assessments were performed before RT and in yearly intervals thereafter. Following an eight-hour fasting, the serum samples were obtained soon in the morning, and the serum basal GH and GH levels following the oral ingestion of 75 g of glucose (OGTT) at 0, 60, 90, and 120 min were assessed with immunoradiometric assay (Immunotech Assays, Marseille, France). The test's sensitivity was 0.1 μg ⁄ L, the intraassay coefficient variation was ≤ 1.5%, and the interassay coefficient variation was ≤ 14%.

IGF-1 levels were measured by RIA after acid–ethanol extraction (Biochem Immunosystem, Freiburg, Germany), with an interassay variation coefficient of ≤ 11% and the following age-corrected normal range: ≤ 460 μg/L (20–30 years), ≤ 360 μg/L (31–40 years), ≤ 310 μg/L (41–50 years), and ≤ 255 μg/L (> 50 years). MRI was obtained at least every 2 years after RT in all patients.

### 2.5. Statistical Analysis

The distribution of data regarding normality was assessed through the Kolmogorov–Smirnov test. Quantitate variables were reported as mean (SD) or median (Q25–Q75), and categorical variables were presented as frequency (percent). A comparison of pre- and post-RT hormone levels is conducted by the Wilcoxon signed-rank test. Analysis was done through the SPSS software (Version 26.0; SPSS Inc., Chicago, IL, USA). The levels of significance are considered *p* values < 0.05.

## 3. Results

### 3.1. Patient Population

The study analysis included 55 patients who underwent conventional RT treatment after transsphenoidal or transfrontal surgery. The average age of participants is 47.09 ± 1.91 years at the time of RT. Of 55 patients who participated in the study, 31 were females (56.4%) and 24 were males (43.6%). All patients had been followed for 2 years, 54 (98%) for 5 years, 50 (90%) for 10 years, and 25 (27%) for 15 years. The median duration of follow-up after RT is 15 (13.3–16.7) yr. Before RT, basal GH levels were 20.7 (16–25.4) μg/L, and IGF-1 concentration was 559.8 (510–609.8) μg/L. Patients were mostly treated with bromocriptine (59.6%), SSAs (21.15%), CAB (11.5%), and PEG (7.6%) after RT during follow-up periods. No patients received combined therapy; all patients received drugs after RT. There were no patients who stopped drug treatment after RT (Table 1).

Three patients were not followed in the research: one died within the follow-up phase because of stroke, one cancer, and one myocardial infarction 5, 10, and 12 years following RT, respectively.

### 3.2. Effect of RT on GH, IGF-1, and Tumor Size

Table 2 illustrates the proportion of cured patients with the GH and IGF-1 normalization rate at 2, 5, 10, and 15 years of follow-up after RT. The basal GH < 2·5 μg/L was reached in 10% of patients at 2 years, 27% of patients after 5 years, and 46% and 84% of participants after 10 and 15 years, respectively. Furthermore, 2 years after RT, suppressed GH < 1 µg/L was seen in 9% of patients, 25% after 5 years, 42% after 10 years, and 76% after 15 years. Also, age-adjusted IGF-1 decreased to the normal range in 5% of participants after 2 years, 14% after 5 years, 38% after 10 years, and 60% after 15 years. Furthermore, RT changed the tumor size (width and length) of acromegaly patients after about 15 years of follow-up relative to baseline values, which include 15 mm (5–25) vs. 20 mm (13.75–25) width (*p*=0.008) and 20 mm (10–25) vs. 25 mm (18.75–25) length (*p*=0.005).

### 3.3. Long-Term Effect of RT on GH and IGF-1 Concentration

**Figure 1:**
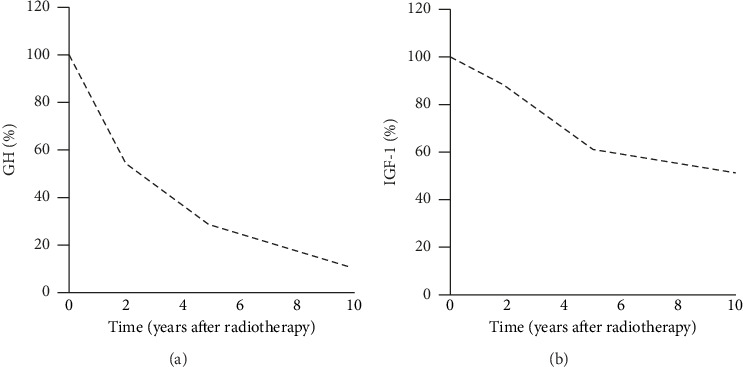
Long-term effects of radiotherapy on GH (a) and IGF-1 (b) levels. Data are represented as mean percentage of GH and IGF-1 relative to preradiotherapy levels.

The effects of radiotherapy on GH and IGF-1 reductions are shown in Figure 1. Basal GH concentration declined by 54%, from 20.7 (16–25.4) µg/L to 11.2 (8.3–14.1) µg/L at 2 years (*p* < 0.001). At 5 years, GH declined by 28% to 5.8 (4.99– 6.61) µg/L (*p* < 0.001), and at 10 years, GH declined by 10.6% to 2.2 (1.66–2.74) µg/L (*p* < 0.001) (Figure 1(a)). IGF-1 concentration decreased from preradiotherapy values of 559 (510–609.8) µg/L by 87% to 490 (434.5–545.5) µg/L at 2 years (*p* < 0.001). At 5 years, IGF-1 declined by 61% to 345 (291–399) µg/L (*p* < 0.001), and at 10 years, by 51% to 285 (230.39–339.61) µg/L (*p* < 0.001) (Figure 1(b)).

### 3.4. Side Effects of RT and Complications

Table 3 shows the prevalence of hypopituitarism in 55 patients with acromegaly treated with RT. Hypopituitarism is the most common adverse effect of RT. Before RT, gonadotropic inadequacy was reported in 49% of the patients. However, thyrotropic inadequacy and corticotropic inadequacy were presented in 25% and 29% of the patients, respectively. The prevalence of hypopituitarism enhanced dramatically following RT; 2 yr after pituitary RT, gonadotropic inadequacy was observed in 64% as well as thyrotropic deficiency, and corticotropic deficiency was observed in 60% of the patients. Ten years after RT, 78% of the patients had gonadotropic deficiency, thyrotropic insufficiency was observed in 80% of the cases, and corticotropic deficiency was present in 82%. In 4% of patients, neurological complications were observed 10 years after RT, which includes potential cerebral necrosis related to the RT; 4% developed visual impairment and optic neuropathy at 5 years of RT.

## 4. Discussion

Acromegaly is distinguished by oncoming somatic defacement, which mostly includes the face, extremities, and other organs and is related to systemic demonstrations. The disease results in the inordinate generation of GH. Acromegaly management includes surgery, medical therapy, and RT and aims to reduce the GH and IGF-1 levels, manage tumor volume, decrease the risk of creating systemic concomitant disease, and decrease mortality [14, 34, 35].

Although medical therapy is proposed to be used in postoperative active acromegaly patients, it is not considered a lifelong treatment because of its high costs in low-income countries.

Nowadays, radiation therapy is regarded as an efficient adjuvant approach in acromegaly treatment and is mainly being used in patients who cannot control the GH and insulin-like growth factor-1 properly or develop tumor growth, even with surgery or medications. The effectiveness of RT on hormonal decrement is demonstrated in different investigations. Various investigations declared in the last decade that RT contributes to an amelioration rate of 40%–70% within 10 years, which consists of a GH value < 2 μg/L and a normal age- and gender-adjusted IGF-1 [36]. However, in our research, we intended to reevaluate the efficacy of RT with precise criteria and cutoffs (basal < 2.5 μg/l GH and glucose-suppressed GH values < 1 µg/L).

In the present study, glucose-suppressed GH values < 1 μg/L accrued in 25%, 42%, and 76% of patients 5, 10, and 15 years following RT, respectively. Furthermore, basal GH < 2.5 μg/L was reached in 27%, 46%, and 84% of participants after 5, 10, and 15 years following RT. According to the results, both indices (basal GH and glucose-suppressed GH) can be used to assess the efficacy of RT in acromegaly patients.

Measurement of the post-OGTT GH level is a recognized index for assessing the effects of interventions in acromegaly. Since basal GH levels of plasma vary considerably, oral glucose loading prevents secretory bursts of GH [37]. On the other hand, IGF-1 decreased progressively in about 14%, 38%, and 60% of patients at 5, 10, and 15 years after RT. IGF-1 concentrations decreased slowly relative to basal GH and post-OGTT GH after RT.

However, there is criticism that RT rarely normalizes IGF-1. In other investigations, the rate of normalization of IGF-1 after conventional RT is reported to be 60%–70% at 10 years [1]. Nowadays, IGF-1 indicates that GH production is applied as an essential scale of remission and presents the outcome of the medical interventions in acromegaly [38, 39]. The data on IGF-1 follow-up after radiotherapy show variation due to differences in follow-up duration, disease status, therapies administered before radiotherapy, the use of different IGF-1 assays, and the varying normal cutoffs used for IGF-1 levels [40]. The most important and common side effect of conventional RT is hypopituitarism, which occurs in 40%–80% of patients at 10 years. In this experience, the side effects associated with RT were neurological complications and visual impairment, including optic neuropathy and a high prevalence of hypopituitarism, which is better to take into consideration when RT is the choice of treatment.

The study is limited by missing data in some cases and by its retrospective design and small sample size, which may influence the results. The main strength points of this investigation are the long-term follow-up of 15 years and performing the surgery and RT protocol by the same neurosurgeon and radiotherapist at one center, leading to more homogenous outcomes and similar follow-up procedures.

## 5. Conclusion

In summary, conventional radiotherapy plays a key role in sustained control of acromegaly symptoms after pituitary surgery for patients not adequately managed by pharmacotherapy. It is efficient in the remission of suppressed GH levels/IGF-1 levels after 15 years of follow-up. We presume that radiation might be an important adjuvant therapy in acromegaly patients uncontrolled by surgery or medical therapy.

## Figures and Tables

**Table 1:** Baseline characteristics of patients.

Variables^∗^	
Age	47.09 ± 1.91

Sex *n* (%)	
Female	31 (56.4)
Male	24 (43.6)

Follow-up after RT (yr)	15 (13.3–16.7)

Number of patients followed for:	
2 years	55 (100)
5 years	54 (98)
10 years	50 (90)
15 years	25 (27)

Tumor size (width-mm)	20 (13.75,25)

Tumor size (Length-mm)	25 (18.75,25)

Preradiotherapy GH basal levels (μg/L)	20.7 (16–25.4)

Preradiotherapy IGF-1 levels (μg/L)	559.8 (510–609.8)

Medical therapy after radiotherapy	
Bromocriptine *n* (%)	31 (59.6)
Somatostatin analogs *n* (%)	11 (21.15)
Cabergoline	6 (11.5)
Pegvisomant	4 (7.6)
Combined drug therapy	0
Not received any drugs	0
Stopped drug treatment	0

Abbreviations: GH, growth hormone; IGF-1, insulin-like growth factor-1.

^∗^Quantitative data are presented as mean ± SD or median (Q25–Q75) and qualitative data are presented as *n* (%).

**Table 2:** Radiotherapy results in acromegaly.

Variables^∗^	2 years	5 years	10 years	15 years
Basal GH (< 2.5 μg/L)	6/55 (10)	15/54 (27)	23/50 (46)	21/25 (84)
GH (after OGTT test) (< 1 µg/L)	5/55 (9)	14/54 (25)	21/50 (42)	19/25 (76)
No. of patients with normal IGF-1 (μg/L)	3/55 (5)	8/54 (14)	19/50 (38)	15/25 (60)

*Note:* Patients with normal GH and IGF-1 concentration.

^∗^The percentages of cured patients are shown at 2, 5, 10, and 15 yr after radiotherapy.

**Table 3:** Prevalence of hypopituitarism in patients with acromegaly treated with radiotherapy (RT).

Pituitary function	Pre-RT *n* = 55	Post-RT			
		2 years *n* = 55	5 years *n* = 54	10 years *n* = 50	15 years *n* = 25
Gonadotropic inadequacy	27 (49%)	35 (64%)	37 (68%)	39 (78%)	15 (60%)
Thyrotropic inadequacy	14 (25%)	33 (60%)	35 (64%)	40 (80%)	16 (64%)
Corticotropic inadequacy	16 (29%)	33 (60%)	37 (68%)	41 (82%)	18 (72%)

*Note:* Data are presented as *n* (%).

## Data Availability

The datasets analyzed in this study are accessible from the corresponding author upon reasonable request.
